# Improvement of Psoriasis by Alteration of the Gut Environment by Oral Administration of Fucoidan from *Cladosiphon Okamuranus*

**DOI:** 10.3390/md18030154

**Published:** 2020-03-10

**Authors:** Masanobu Takahashi, Kento Takahashi, Sunao Abe, Kosuke Yamada, Manami Suzuki, Mai Masahisa, Mari Endo, Keiko Abe, Ryo Inoue, Hiroko Hoshi

**Affiliations:** 1 Department of Biotechnology, Maebashi Institute of Technology, 460-1 kamisadori-machi, maebashi, Gunma 371-0816, Japan; mt1181028@gmail.com (M.T.); kento11240504@gmail.com (K.T.); yg26utumi@gmail.com (K.Y.); manami_bird@yahoo.co.jp (M.S.); collon.mmmf@gmail.com (M.M.); kuoria112@yahoo.co.jp (M.E.); 2 Marine Products Kimuraya Co., Ltd., 3307 Watari-cho, Sakaiminato, Tottori 684-8790, Japan; abe@mozuku-1ban.jp; 3 Graduate School of Agricultural and Life Science, The University of Tokyo, 1-1-1 Yayoi, Bunkyo-ku, Tokyo 113-8657, Japan; aka7308@mail.ecc.u-tokyo.ac.jp; 4 Group of Food Functionality Assessment, Kanagawa Institute of Industrial Science and Technology, Kawasaki-ku, Kawasaki, Kanagawa 213-0012, Japan; 5 Department of Agriculture and Life science, Kyoto Prefectural University, 1-5 Shimogamohangi-cho, Sakyo-ku, Kyoto 606-8522, Japan; r-inoue@kpu.ac.jp

**Keywords:** fucoidan, psoriasis, *Traf3ip2*, microbiota, mucin, IgA

## Abstract

Psoriasis is a chronic autoimmune inflammatory disease for which there is no cure; it results in skin lesions and has a strong negative impact on patients’ quality of life. Fucoidan from *Cladosiphon okamuranus* is a dietary seaweed fiber with immunostimulatory effects. The present study reports that the administration of fucoidan provided symptomatic relief of facial itching and altered the gut environment in the TNF receptor-associated factor 3-interacting protein 2 (*Traf3ip2*) mutant mice (*m-Traf3ip2* mice); the *Traf3ip2* mutation was responsible for psoriasis in the mouse model used in this study. A fucoidan diet ameliorated symptoms of psoriasis and decreased facial scratching. In fecal microbiota analysis, the fucoidan diet drastically altered the presence of major intestinal opportunistic microbiota. At the same time, the fucoidan diet increased mucin volume in ileum and feces, and IgA contents in cecum. These results suggest that dietary fucoidan may play a significant role in the prevention of dysfunctional immune diseases by improving the intestinal environment and increasing the production of substances that protect the immune system.

## 1. Introduction

Psoriasis is a chronic autoimmune inflammatory disease characterized by skin lesions and abnormal keratinocyte proliferation. The number of cases of psoriasis vulgaris is increasing worldwide [1]. The etiology of psoriasis remains unclear, although there is evidence for genetic predisposition. The TNF receptor-associated factor 3-interacting protein 2 (*TRAF3IP2*) gene was identified in 2010 as a new susceptibility locus containing the psoriasis vulgaris disease gene in European genome-wide association studies [2]. The *TRAF3IP2* gene encodes a protein involved in IL-17 signaling, which interacts with members of the nuclear factor-kappa-B transcription factor family. Recently, an important genetic influence of the polymorphism in *TRAF3IP2* on the susceptibility to psoriasis, but not to atopic dermatitis, was reported in a Japanese population [3,4]. Matsushima et al. [5] also reported that a genetic mutation in *Traf3ip2* mice caused an atopic dermatitis-like skin disease with hyper-IgE-emia. 

The gut microbiota observed in patients with psoriatic was less diverse when compared to that of healthy controls [6]. The gut microbiota profile in the gut environment has been found to significantly influence autoimmune diseases such as multiple sclerosis [7,8]. Recently, however, adults with psoriasis and/or psoriatic arthritis have learned to supplement their standard medical therapies with dietary interventions to reduce disease severity [9]. There are many reports about the relationship between immunostimulatory effects and dietary components of microbiota [10]. Polysaccharides are considered a dietary fiber, and work as prebiotics which are beneficial for the intestinal environment [11]. Fructo-oligosaccharides maintain intestinal barrier function, as does immunoglobulin A (IgA), in a methionine–choline-deficient mouse model of nonalcoholic steatohepatitis [12]. Secreted IgA cells in colonic tissue generate mucus that is secreted into the outermost intestinal cecal patch; the secreted mucus entraps bacteria and prevents their translocation into the tissue [13].

This study was focused on fucoidan, which is found in the cell wall matrix of brown algae. Fucoidan is a high-molecular weight (over 200,000 Daltons) sulfated polysaccharide; it consists mostly, i.e., 13% or more, of sulfated fucose and glucuronic acid [14,15]. Many reports have reported that fucoidans from various brown algae have some biological activities, such as antitumor, anticoagulant, and apoptosis induction, along with other antiallergic and immunologic activities [16,17,18]. Fucoidan supplementation improved fecal microbiota composition and repaired intestinal barrier function; moreover, fucoidan is an intestinal flora modulator for the potential prevention of breast cancer [19]. However, it has not been reported whether fucoidan in the diet affects psoriasis or changes the intestinal environment components of the intestinal environment such as mucin, IgA, and bacterial flora. 

The present study demonstrated that fucoidan affects psoriasis symptoms; it ameliorated the effect of phenotype and altered the intestinal microbiota and the quality of secreted IgA and mucin in m-*Traf3ip2* mutant mice.

## 2. Results

### 2.1. Fucoidan Diet Rescued Psoriasis Symptoms on m-Traf3ip2 Mouse Faces and Reduced Scratching

**Figure 1:**
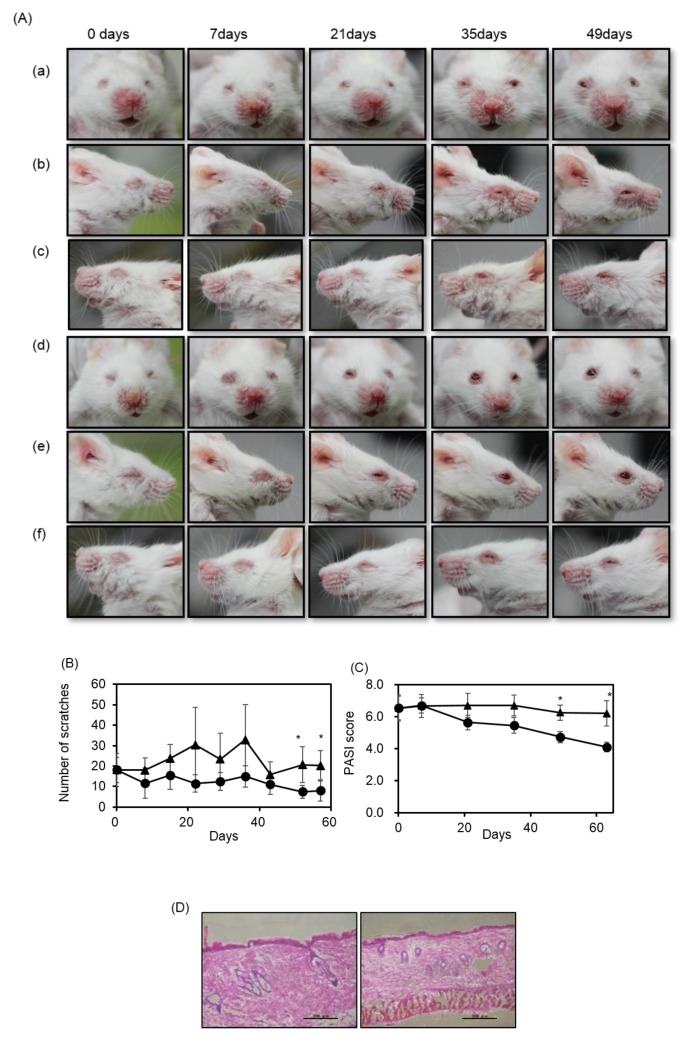
Effects of fucoidan diet on *m-Traf3ip2* mouse faces by PASI score analysis and scratching actions. (**A**) Aspects of *m-Traf3ip2* typical mouse face over time. (a), (b), (c) Sections are normal diet mice and (d), (e), (f) sections are fucoidan diet mice. (**B**) Scratching behavior of individual mice. Closed triangle shows normal diet group and closed circle shows fucoidan diet group. PASI test scored 5 persons and each value. The calculated values for the in vitro experiments are mean ± SD (fucoidan diet group *n* = 14 and normal diet group *n* = 9). (**C**) The severity of the psoriasis-like skin condition. (**D**) Histological analysis by HE staining of faces. Normal diet mice and fucoidan diet mice epidermal sections are shown on left and right, respectively. Bars show 0.2 mm. Asterisk (*) shows significant difference (*p* < 0.05).

Psoriasis is a chronic immunological inflammatory disease of the skin characterized by dry silvery scales with eruptions. In this study, the severity of symptoms on the faces of *m-Traf3ip2* mice was graded by phenotype using a scoring system based on clinical Psoriasis Area and Severity Index (PASI) and an ethological method as a scratch test. For this study, 1% fucoidan or 1% cellulose was administered by adding it to the AIN-93G diet, which is a normal diet. *m-Traf3ip2* mice facial skin showed significant differences in symptoms between the fucoidan and normal diet groups. *m-Traf3ip2* mice which were on the normal diet showed severe symptoms of psoriasis on their faces, whereas the mice on the fucoidan diet showed improved facial symptoms after 21 days and tended to enter remission in the following weeks (Figure 1A). The normal diet group exhibited increased scratching within a certain time frame, whereas the fucoidan diet group showed significantly decreased facial scratching from 1 week until after 56 days (Figure 1B). In the normal diet group, the mice with the highest PASI scores showed the most serious symptoms and the most severe scratch test results. On the other hand, the symptoms of the fucoidan diet mice group decreased from 21 days until after 63 days, and were significantly lower than in those of the control group (Figure 1C). Histological sections of the facial skin of mice that had high PASI scores showed thickening of both the epithelium and keratin layer of the epidermis by hematoxylin-eosin (HE) staining (Figure 1D). 

These results suggested that fucoidan improved psoriasis symptoms on the faces of *m-Traf3ip2* mice.

### 2.2. Fucoidan Drastically Changed Microbiota in the Small Intestines of m-Traf3ip2 Mice

**Figure 2:**
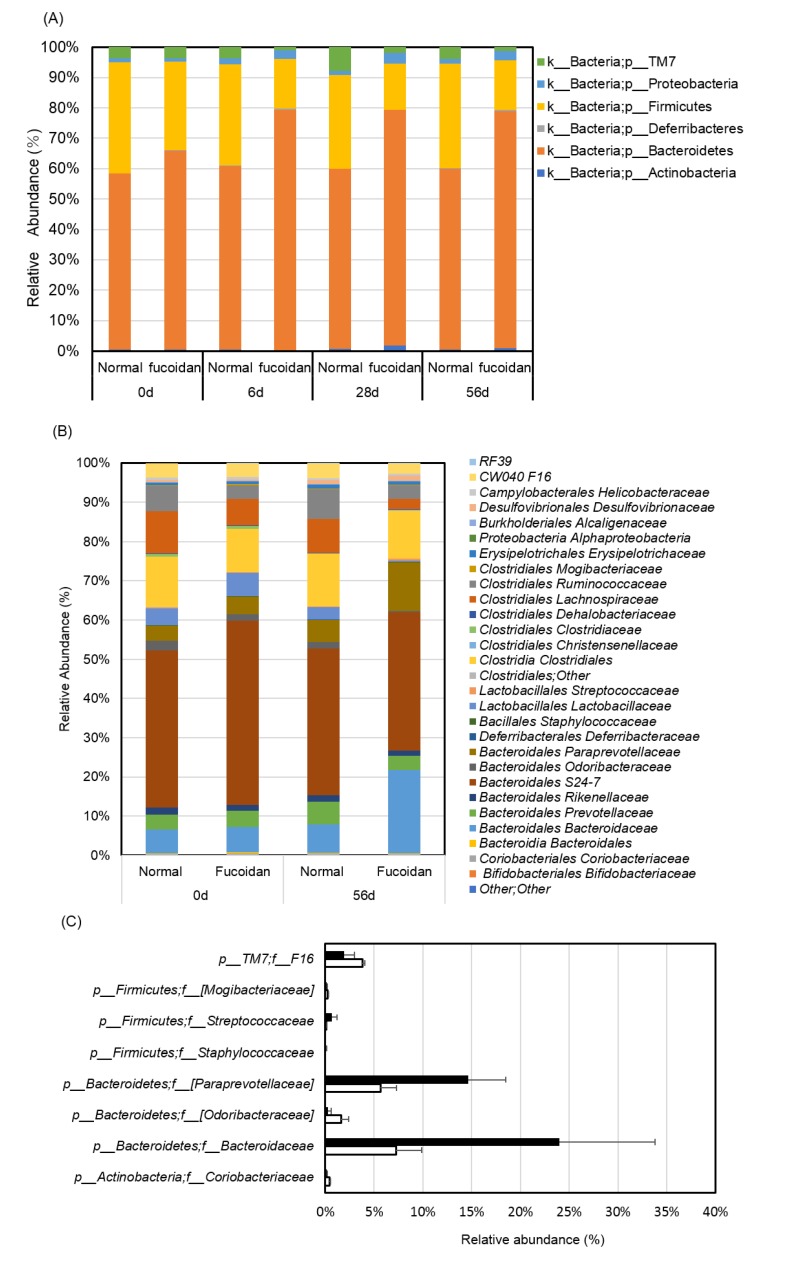

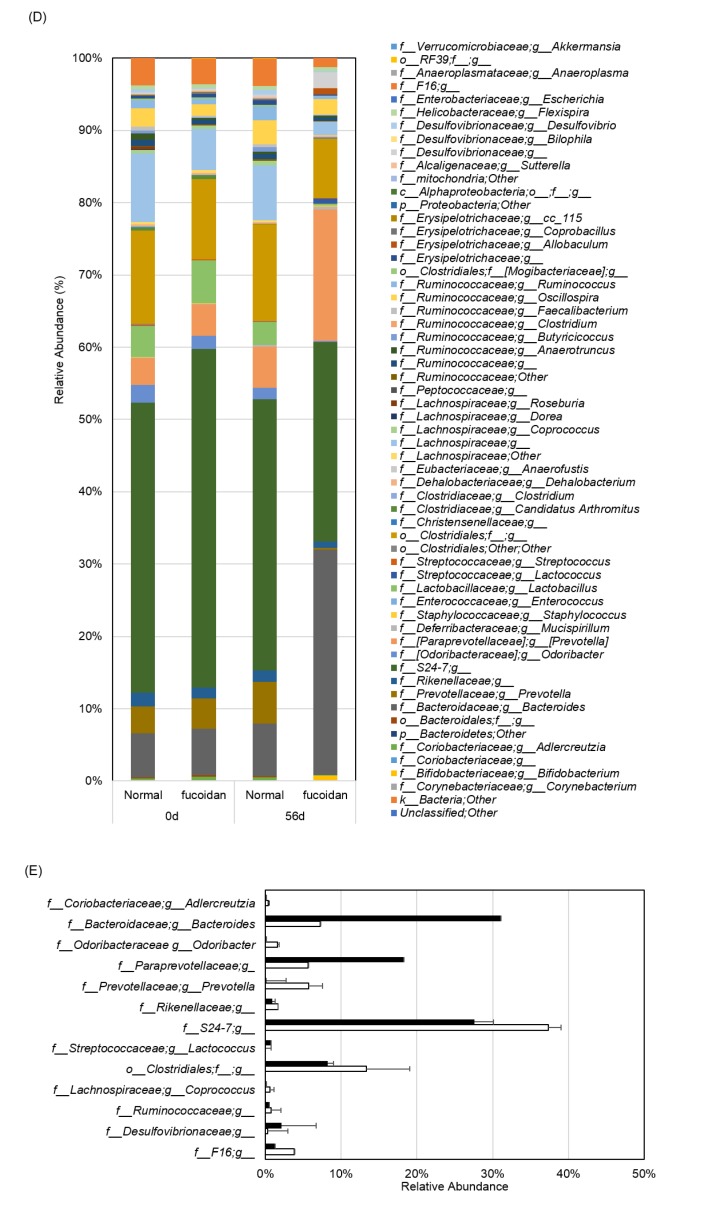
Fecal microbiota analysis. 16S rRNA genome-wide screening analysis. Time dependence of fecal microbiota analyses were performed on the phylum (**A**), family (**B**), (**C**), and genus (**D**), (**E**), levels of normal diet group (left) and fucoidan diet group (right). White bar shows normal diet group and black bar shows fucoidan group. The calculated values for the *in vitro* experiments are means ± SD (*n* =5).

Dietary fiber is a prebiotic, which is useful in the intestinal environment [11,12]. In the present study, 16S rRNA from intestinal microorganisms in fecal samples was analyzed using next-generation genome sequences. In phylum analysis, fecal microflora in the fucoidan diet group showed significantly increasing relative abundance of the *Bacteroidetes* and *Proteobacteria* phyla during each time course (i.e., 6, 28, and 56 days) compared to mice fed the normal diet, whereas the relative abundance of the *Firmicutes* and *TM7* phyla significantly decreased in the fucoidan diet group after 6 days and remained at a low level until the end of the experiment (Figure 2A) compared to the fecal microorganisms of the normal diet fed mice. Table 1 shows the changes in phylum levels in fecal microflora of *m-Traf3ip2* fucoidan diet and normal diet mice over time. The relative abundance of *Deferribacteres* and *Actinobacteria* phyla, which were the lowest of all the phyla, were higher in the fucoidan diet group than in the normal diet group. The differences between the fucoidan and normal diet samples were then analyzed by taxonomy, class, order, family, and genus. After 56 days, levels of fecal microbiota belonging to the *Bacteroidaceae* and *Paraprevotellaceae* families in the *Bacteroidetes* phylum in fucoidan diet group were significantly higher than those of normal diet group. On the other hand, the fecal microbiota of fucoidan diet group in the family of *F16* in the phylum of *TM-7,* and those in the family of *Odoribacteraceae* in the phylum *Bacteroidetes*, showed decreased levels of the same kinds of microflora after 56 days (Figure 2B,C) compared to the microbiota of normal diet mice group. In the genus analysis, fecal microflora of fucoidan diet group showed significantly increased relative abundance of unclassified *Paraprevotellaceae* genera and *Bacteroides* genera in the family of *Bacteroidetes* compared to fecal microflora of the normal diet group (Figure 2D,E). 

Microorganisms of the *Desulfovibrionaceae* family were found at slightly higher levels in the fecal microbiota of the fucoidan diet group compared to that of the normal diet group. The fecal microbiota of fucoidan diet group showed significantly lower populations of the genera *Prevotella* and *Odoribacter*, as well as of an unclassified *S24-7* family in the *Bacteroidetes* phylum, than in the normal diet group after 56 days. In the *Firmicutes* phylum, the genera of *Coprococcus*, unclassified members of the *Ruminococcaceae* family, and unclassified members of the order *Clostridiales* were lower in fecal microbiota of the fucoidan diet group compared to those of the normal diet group. The normal diet group showed significant increase in the fecal microbiota of *F16* family compared to those of the fucoidan diet group. 

These results suggested that the fucoidan diet altered the taxonomic compositions of many microbiota in *m-Traf3ip2* mice 6 days after feeding, and thereafter, the microbiota conditions were continuously maintained. 

### 2.3. Fucoidan Diet Promoted Fecal Mucin Production

**Figure 3:**
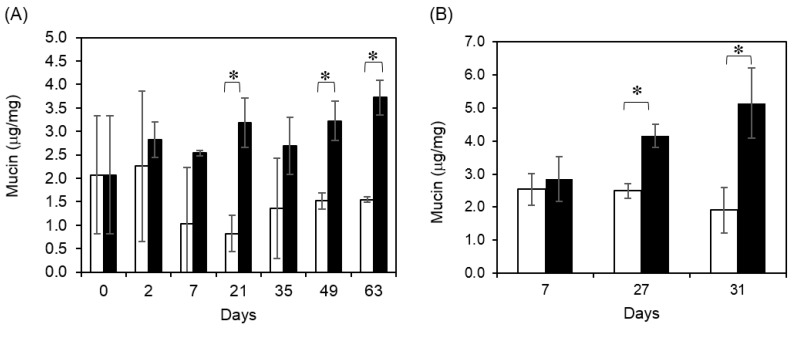
Quantification of mouse fecal mucin. Volume concentrations of (**A**) *m-Traf3ip2* and (**B**) wild type mouse in fecal mucin. White bar shows normal diet group and black bar shows fucoidan group from fecal. The calculated values for the in vitro experiments are mean ± SD (*n* = 5). Asterisk (*) shows significant difference (*p* < 0.05).

Mucin, which is produced by mucous/goblet cells, is a major component of mucosal fluid. Alterations in gastrointestinal mucin induced by dietary fiber may affect nutrient bioavailability, cytoprotection of the mucosa, or other aspects of gastrointestinal function [20,21]. In this study, mucin production in feces by mucous goblet cells was measured. Fluorescence labeling was used to measure terminal N-acetylgalactosamine in mucin components in feces. In *m-Traf3ip2* mice, the fucoidan diet group showed increased mucin production, especially from 21 days after the diet had begun, whereas the normal diet group showed little change (Figure 3A). Similarly, wild-type, not mutated gene of *Traf3ip2* BALB/c mice, fed a fucoidan diet showed significantly increasing mucin secretion compared to the group of a normal diet mice (Figure 3B). These results suggested that fucoidan promoted mucin production and created a better intestinal environment. 

### 2.4. Production of IgA in Feces and Cecum

**Figure 4:**
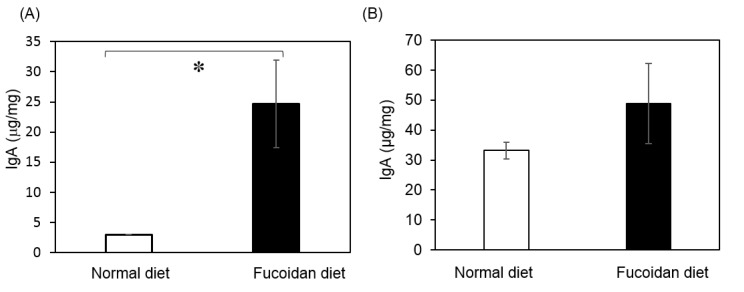
Quantification of total IgA in cecal contents of fucoidan diet group and normal diet group. Volume concentrations of (**A**) *m-Traf3ip2* and (**B**) wild type mouse total IgA were quantified from 63 days and 31 days, respectively, in cecum contents by ELISA. White bar shows normal diet group and black bar shows fucoidan diet group from cecum. The absorbance of reaction products was determined at 450 nm, and the production was quantified. The calculated values for the in vitro experiments are expressed as mean ± SD (*n* = 5). Asterisk (*) shows significant difference (*p* < 0.05).

Secreted of IgA in the gut mucosal layer plays important roles in intestinal immune function, e.g., prevention of bacterial and viral invasion. IgA suppresses the expression of bad bacteria and maintains a healthy balance of gut microbiota. Enzyme-linked immunosorbent assay (ELISA) was used to evaluate IgA expression in feces and cecum of *m-Traf3ip2* mice fed on fucoidan with or without diet for 63 days. The assay showed that secreted IgA was not present, or was present at levels below detection, in fecal samples from both groups. It was reported that the cecum produces IgA [22]. Our study detected total IgA in the cecum of both groups in the samples obtained at 63 days. Total IgA volume was obviously higher in the ceca of the fucoidan fed mice group compared to those of the normal diet mice group (Figure 4A). Wild type mice fed a fucoidan diet also showed higher total IgA compared to the normal diet group of wild type mice (Figure 4B).

This result indicated that fucoidan promotes the secretion of IgA in the cecum and improves the intestinal immunity. 

## 3. Discussion

The present study showed that the administration of the sulfated polysaccharide fucoidan improves the symptoms of psoriasis, an immune disorder disease, changes the composition of intestinal microbiota to include high relative abundance of *Bacteroidetes*, and improves the gut environment by increasing the volumes of mucin and IgA. 

First, the administration of the fucoidan diet to psoriasis model mice with the *m-Traf3ip2* mutation gradually improved symptoms, and especially significantly improved phenotypes, scratch test scores, and PASI scores, beginning at 21 days until after 56 days. However, *m-Traf3ip2* mice did not show a preference for the fucoidan diet, and mice in the two dietary groups showed no weight difference. In the same period, we showed the alteration of microflora and the production of mucin at the lamina propria of the mucous membrane and of IgA in cecum. *Bacteroidetes* and *Firmicutes*, which comprise more than 90% of all phylogenetic types, are the two dominant bacterial phyla in the human, mouse [23,24,25], and pig [26] gut. An analysis of fecal microbiota by genome sequences revealed that feeding of fucoidan to *m-Traf3ip2* mice significantly increased relative abundance of the phylum *Bacteroidetes* for all bacteria beginning at 6 days and continuing beyond 56 days compared to the relative abundance in the normal diet mice. 

In a comparison between two phylum groups, *Bacteroidetes* and *Firmicutes*, for all bacteria, *Firmicutes* levels decreased from 2 days after the beginning of the experiments and remained low after 56 days; the analysis was performed using quantitative PCR. These results showed that feed intake of fucoidan by *m-Traf3ip2* mice increased the relative abundance of the *Bacteroidetes* phylum and decreased those of the *Firmicutes* phylum. However, the family *S24-7* in the phylum *Bacteroidetes* was decreased in the fucoidan diet group. It was reported that *S24-7* is related to bowel inflammation [27]. A fucoidan diet changes fecal microbiota and may also have anti-inflammatory effects. On the other hand, the fucoidan diet group showed drastically decreased the relative abundance of the phylum *Firmicutes*. *Firmicutes* was reported to have a wide range of both beneficial and harmful effects on autoimmune disorders. The feces of fucoidan diet group showed a significantly lower relative abundance of the phylum *Firmicutes* compared with the growth rate of control diet group. In this study, levels of two *Clostridiales* families, *Lachnospiraceae* and *Ruminococcaceae*, were decreased in the intestinal microflora of the fucoidan diet group. Especially, fucoidan administration decreased the presence of *Ruminococcaceae Oscillospira* species in the intestinal microbiota. Our results corresponded with those of another microbiota analysis: soluble dextrin fibers altered the intestinal microbiota and reduced proinflammatory cytokine secretion in male IL-10-deficient mice [28]. Some unidentified members of the *Clostridiales* family may be related to the induction of Th17 cells in the small intestine and aid in protection against pathogens. Th17 cell induction is harmful in patients with an autoimmune disorder [29]. 

The relative abundance of the phylum *Proteobacteria*, family *Desulfovibrionaceae,* was also higher in the fucoidan diet group than in the control group. Glycomacropeptide is a prebiotic that reduces *Desulfovibrio* bacteria, increases cecal short-chain fatty acids, and has an anti-inflammatory effect in mice [30]. In the present study, fucoidan was a possible source of *Desulfovibrionaceae* consumption as a source of nutrition. The increased relative abundance of the family *Desulfovibrionaceae* may be related to some improvement in the symptoms of psoriasis in the present study. The analysis suggested that the fecal microbiota of *Bacteroidetes acidifaciens* were drastically increased in fucoidan diet group in this study (data not shown). Species of *B. acidifaciens* promote the expression of secreted IgA in the large intestine [13,31], and members of the genus *Rikenellaceae* are often found in the gastrointestinal tracts of a number of animals [32]. The species of *B. acidifaciens* may be related to the improvement of symptoms of psoriasis. Additionally, levels of the *Bacteroidetes* species *Parabacteroides* was increased in the fecal microbiota of the fucoidan diet group. Intestinal microbiota of the genus *Bacteroides*, as well as those of *Porphyromonadaceae Parabacteroides*, produce antagonistic substances in ecological niches, preventing the colonization and invasion of exogenous bacteria, and might be one of the mechanisms underlying such prevention [33]. In the present study, drastic increase in the volumes of cecal IgA and ileal mucin was observed in the intestines of fucoidan fed *m-Traf3ip2* mice. Interestingly, these increases coincided with the increase in the *Bacteroidetes* population. β-glucans also have distinctive immunomodulatory characteristics [11,34,35]. In children with chronic respiratory problems, short-term oral application of β-glucan affects mucosal immunity by stabilizing secreted IgA levels [36]. The present results suggest that fucoidan administration induces the production of IgA and mucin, rearranging the intestinal environment to regulate immune response [37]. In pigs, a laminarin diet affects immune function by increasing genes that encode mucin expression, namely, *MUC2*, *MUC4*, IL-6, and IL-8 in the ileum [11]. Laminarin also binds to mammalian non-Toll-like pattern-recognition receptors, such as, dectin-1, complement receptor-3, lactosylceramide, and scavenger, thereby stimulating innate immunity through the activation of macrophages, dendritic cells, neutrophils, natural killer cells, and helper T-cells [35]. Our study did not elucidate the relation of administration of fucoidan and its molecular action in psoriasis model mice. It will be necessary to elucidate the mechanism underlying the effect of fucoidan on the remission of psoriasis. 

In conclusion, fucoidan as a dietary supplement could modulate the fecal microbiota composition and repair intestinal barrier function. This study suggested that fucoidan can be used to improve the symptoms of psoriasis.

## 4. Materials and Methods 

### 4.1. Reagents

All reagents used in the enzyme assays were obtained from Wako Pure Chemical Industries (Tokyo, Japan). Fucoidan from *Cladosiphon Okamuranus* was provided by Marine Products Kimuraya Co., Ltd. (Tottori, Japan). The average molecular weight of fucoidan is 300,000 Dalton as analyzed by size exclusion chromatography. The fucose content is about 60%, and sulfated fucose is 14.3% in total fucoidan. Food entrainment, i.e., AIN-93G, was purchased from CREA Japan Inc. (Tokyo, Japan). 

### 4.2. Animals and Diets

*m-Traf3ip2* and BALB/c mice were previously generated as described in prior studies [5] and purchased from Japan SLC, Inc. (Shizuoka, Japan). SPF-grade animals were maintained under conventional conditions in animal facilities certified by the Animal Care and Use Committee of the Maebashi Institute of Technology. The experimental procedures were all in accordance with the National Institutes of Health guidelines for the care of experimental animals, and the experimental protocol was approved by the Institutional Animal Care and Use Committee of Maebashi Institute of Technology (16-001). The animals were housed individually in plastic cages, allowing for the separation and collection of feces, at 21 °C with a relative humidity of 55% under a 12:12 h light:dark cycle. 6-week-old mice were housed as 5 mice per cage in a controlled environment. After 1 week of acclimatization, the mice were randomly divided into two groups, and thereafter, were fed the experimental/normal or fucoidan diets with free access to drinking water. The animals were fed Rodent Diet AIN-93G (CLEA Japan, Tokyo, Japan). Two groups of 14 or 9 *Traf3ip2* mutant mice were fed a diet containing 1.0% w/w fucoidan or crystalline cellulose, respectively. Each group was fed for 9 weeks. Two groups of five wild type, BALB/c mice, were fed a diet containing 1.0% w/w fucoidan or crystalline cellulose, respectively. Each group was fed for 31days.

### 4.3. Analysis of Phenotype and Scratch Test

The face of each mouse was photographed from three angles: front, left side, and right side. To score the severity of skin inflammation, the severity of the psoriasis-like skin condition in the facial area of *m-Traf3ip2* mice was monitored and graded every 7 days. The scoring system was based on the clinical PASI [38]. The modified PASI score was based on three parameters, namely, erythema, scaling, and thickening. Each parameter score was assigned independently on a scale of 0 to 4 as follows: 0, none; 1, slight; 2, moderate; 3, marked; and 4, very marked. A scoring table with red taints was used to score the level of erythema. The cumulative score (erythema, desquamation, swelling, and scaling area) denoted the severity of inflammation (scale 0–100%). The modified PASI test was applied to each mouse by five persons. Accurate scratching behavior of individual mice was assessed by counting the scratches. Scratching behavior was monitored and the number of scratching movements was counted during a 10-minute period each week.

### 4.4. Histochemical Analysis of Facial Skin Dissection 

After shaving the faces of mice, their facial skin was fixed with 4% paraformaldehyde and then OTC-embedded punch biopsies were sectioned longitudinally into 7–8 μm thick sections. The sections were stained using HE for histological evaluation and for the evaluation of the microarchitecture of thickened epithelium and the keratinocyte layer. Experiments were conducted in triplicate; the data were averaged to evaluate inflammation and epithelial and keratinocyte changes.

### 4.5. Microbiota Analysis in Feces

DNA was extracted from fecal samples for microflora analyses using 16S rRNA high-throughput sequencing. For DNA extraction, the fecal microbiomes were analyzed from 20 mg fecal samples obtained from each animal in the normal as well as fucoidan diet group. For each mouse in either group, total DNA was extracted from a 100 mg fecal sample using QIAamp DNA Stool Mini Kit (Qiagen, Venlo, Netherlands) according to the manufacturer’s instructions. The DNA concentration was determined using a NanoDrop (Scrum, Tokyo, Japan). Extracted fecal DNA was examined using 16S rDNA gene sequencing by MiSeq (Illumina, Tokyo, Japan). Library preparation, deep sequence, and data analysis were carried out using methods described by Inoue et al [39]. Data analysis was performed by Bio-Linux, a Linux computing platform customized for bioinformatics research [40].

### 4.6. Quantification of Fecal Mucin and Cecum IgA

Mucin was extracted from each 100 mg fecal sample. Fecal mucin contents were determined using a fecal mucin assay kit (Mucin Assay Kit, Cosmo Bio, Co., Ltd., Tokyo, Japan). A fluorometric assay discriminated O-linked glycoproteins (mucins) from N-linked glycoproteins. Fluorescence was measured using a SoftMax^®^ Pro spectrometer (Molecular Devices, CA, USA). Total IgA was extracted from 50 mg of cecum samples and quantified using a mouse IgA ELISA quantitation kit (Immundiagnostik, Bensheim, Germany) as specified in the manufacturer’s instructions. The reaction products were determined from absorbance at 450 nm using XFluor4 (Tecan, Zurich Switzerland), and IgA was qualified. 

### 4.7. Statistical Analysis 

The calculated values for the in vitro and in vivo experiments are expressed as mean ± SD. Student’s *t* test was used for statistical analysis.

## Figures and Tables

**Table 1:** Changes in phylum levels in fecal microbiota of *m-Traf3ip2* fucoidan diet group and normal diet group. Significant difference between fucoidan and normal diets. (*p* < 0.01 (c), *p* < 0.05 (d)). Significant difference from before to after same diet administration. (*p* < 0.01 (a), *p* < 0.05 (b)).

Administration Days (D)	0 D		6 D		28 D		56 D	
Diet	Norml	Fucoidan	Normal	Fucoidan	Normal	Fucoidan	Normal	Fucoidan
Relative abundance (%)								
Actinobacteria	0.43 ± 0.15	0.48 ± 0.15	0.38 ± 0.04	0.25 ± 0.05 _(b, d)_	0.70 ± 0.19	1.88 ± 1.52 _(b, c)_	0.45 ± 0.15	0.83 ± 0.72
Bacteroidetes	60.0 ± 7.69	65.4 ± 5.57	60.2 ± 2.90	79.2 ± 1.74 _(c, a)_	59.1 ± 4.18	77.4 ± 2.78	59.4 ± 9.69	78.2 ± 6.42 _(b, d)_
Deferribacteres	0.10 ± 0.07	0.08 ± 0.04	0.25 ± 0.11	0.33 ± 0.33	0.05 ± 0.05	0.10 ± 0.00 _(a, c)_	0.20 ± 0.12	0.40 ± 0.31
Firmicutes	36.3 ± 8.56	29.2 ± 4.43	33.1 ± 2.18	16.3 ± 2.25 _(b, c)_	30.8 ± 2.27	15.2 ± 1.9 _(a, c)_	34.3 ± 9.05	16.3 ± 4.98 _(b, d)_
Proteobacteria	1.35 ± 0.27	1.10 ± 0.16	1.95 ± 0.30	3.03 ± 0.33 _(c, a)_	1.40 ± 0.27	3.50 ± 0.87 _(a, c)_	1.73 ± 0.53	3.05 ± 0.62 _(a, d)_
TM7	3.75 ± 1.51	3.58 ± 1.29	3.73 ± 0.62	0.98 ± 0.26 _(b, c)_	7.78 ± 1.92	1.98 ± 0.19 _(c)_	3.80 ± 0.24	1.23 ± 0.11 _(b, c)_
